# Metagenomes and Metagenome-Assembled Genome Sequences from Nitrogen-Fixing Alder Nodules

**DOI:** 10.1128/mra.01266-22

**Published:** 2023-04-04

**Authors:** Neslihan Taş, Nancy Conejo, Verity G. Salmon

**Affiliations:** a Earth and Environmental Sciences Area, Lawrence Berkeley National Laboratory, Berkeley, California, USA; b Biosciences Area, Lawrence Berkeley National Laboratory, Berkeley, California, USA; c Environmental Sciences Division and Climate Change Science Institute, Oak Ridge National Laboratory, Oak Ridge, Tennessee, USA; University of Maryland School of Medicine

## Abstract

Bacterial nitrogen (N) fixation in alder nodules is a key process providing nitrogen to nutrient-limited arctic biomes. Here, 45 prokaryotic metagenome-assembled genome (MAG) sequences from root nodules of arctic alder are reported.

## ANNOUNCEMENT

Plant roots are home to beneficial microbes where microorganisms help plants to obtain nutrients, tolerate stress, and sustain growth (1). We investigated the nodule microbiome of Alnus viridis subsp. *fruticosa*, an N-fixing deciduous alder shrub that is an important contributor to N cycling in high-latitude ecosystems (2).

Alder nodules were collected from the Next Generation Ecosystem Experiments Arctic Kougarok site (65°09′50.1″N, 164°49′34.2″W), which is located 103 km from Nome, Alaska. At this location, alder is found in two distinct communities, short and dispersed alder growing in lowland areas (savanna) and tall alder growing as dense shrublands along the hillslope (shrubland) (3). Root nodules were collected from five randomly selected plants for each alder type, stored on blue ice in the field, and frozen at −20°C within 10 hours of collection. Nodule surfaces were cleaned before DNA extraction (4), and surface cleanness was confirmed by plating on LB and R2A agar.

Genomic DNA was extracted from each nodule by Qiagen DNeasy DNA isolation kit (Qiagen, MD, USA) (5). DNA from individual nodules was pooled for each plant type and concentrated with Zymo DNA Clean & Concentrator-5 kit (Zymo Research, CA, USA). Two pools of DNA, one from savannah and another from shrubland, were quantified using the Qubit double-stranded DNA high-sensitivity assay (Invitrogen, CA, USA) and submitted for sequencing. Libraries were prepared according to the manufacturer’s instructions with Kapa HyperPrep kit (Roche, CA, USA) and sequenced with Illumina NovaSeq 6000 S4 at QB3 UC Berkeley, generating sequences of 150 bp in length. Both samples had average GC content of 63% and a similar number of paired-end sequences (shrubland, 2.08 × 10^8^ sequences/sample; savanna, 2.19 × 10^8^ sequences/sample). Sequence processing and metagenome-assembled genome (MAG) generation were performed via metaWRAP v1.1 (6). Default parameters were used for all software unless otherwise specified. Sequencing adapters and low-quality reads were removed with metaWRAP::Read_qc module. Reads from both metagenomes were combined into a single set and then coassembled with MEGAHIT v1.1.3 (7), resulting in 475,302 contigs (maximum, 506,155 bp; average, 2,868 bp; *N*_50_, 3,779 bp) of ≥1 kb. Contigs were binned with MaxBin2 v2.2.5 (8), MetaBAT2 v2.12.1 (9), and CONCOCT v1.1.0 (10) and then refined into MAGs using metaWRAP::Bin_refinement module (8). We used CheckM v1.1.10 (11) to determine the completeness and contamination of MAGs. We recovered 6 high-quality and 39 medium-quality draft MAGs (see Table 1 at https://tinyurl.com/4dtt38mt) by minimum information about a metagenome-assembled genome (MIMAG) standards (12). GTDB-Tk v1.5.0, (13) with the reference database GTDB R06-RS202 (14), was used to assign taxonomy where MAGs were distributed across the following phyla: *Proteobacteria*, 25; *Actinobacteria*, 4; *Bacteroidetes*, 8; *Acidobacteria*, 4; *Verrucomicrobia*, 4; and *Myxococcota*, 2. These data provide a rare resource to be analyzed with measurements of plant productivity (3) traits to link N fixation, alder nodule microbiome, and plant health in the changing Arctic ecosystems.

### Data availability.

MAGs were deposited in the National Center for Biotechnology Information (NCBI) BioProject database under accession no. PRJNA830531, and nodule metagenomes were deposited under SRA accession no. SRR18933204 for alder savanna and SRR18933205 for alder shrubland. GenBank accession numbers for the MAGs are listed in Table 1, and quality metrics are provided via Table 1 at https://figshare.com/articles/dataset/Quality_metrics_for_metagenome-assembled_genomes_of_Alnus_viridis_spp_Fruticose_nodules/22096520/1.

**TABLE 1:** Metagenome assembled genomes from Alnus viridis subsp. *fruticose* nodules

Bin ID	GenBank accession no.	MIMAG bin quality^*a*^	GTDB lineage
NGEE_A_bin1	JAMFTE000000000	MQ	p_Proteobacteria;c_Gammaproteobacteria;o_Steroidobacterales;f_Steroidobacteraceae;g_;s_
NGEE_A_bin10	JAMFTD000000000	MQ	p_Proteobacteria;c_Gammaproteobacteria;o_Xanthomonadales;f_Rhodanobacteraceae;g_Rhodanobacter;s_Rhodanobacter sp014207185
NGEE_A_bin11	JAMFTC000000000	MQ	p_Proteobacteria;c_Alphaproteobacteria;o_Azospirillales_A;f_BOG-932;g_BOG-932;s_
NGEE_A_bin12	JAMFTB000000000	MQ	p_Acidobacteriota;c_Acidobacteriae;o_Acidobacteriales;f_Acidobacteriaceae;g_Terriglobus;s_
NGEE_A_bin13	JAMFTA000000000	MQ	p_Proteobacteria;c_Alphaproteobacteria;o_Caulobacterales;f_Caulobacteraceae;g_CAIOYC01;s_
NGEE_A_bin14	JAMFSZ000000000	MQ	p_Proteobacteria;c_Gammaproteobacteria;o_Steroidobacterales;f_Steroidobacteraceae;g_Bog-1198;s_
NGEE_A_bin15	JAMFSY000000000	HQ	p_Verrucomicrobiota;c_Verrucomicrobiae;o_Methylacidiphilales;f_UBA1321;g_JABDGH01;s_
NGEE_A_bin16	JAMFSX000000000	MQ	p_Proteobacteria;c_Alphaproteobacteria;o_Acetobacterales;f_Acetobacteraceae;g_CAIVDX01;s_
NGEE_A_bin17	JAMFSW000000000	MQ	p_Proteobacteria;c_Gammaproteobacteria;o_Burkholderiales;f_Burkholderiaceae;g_LMUX01;s_
NGEE_A_bin18	JAMFSV000000000	MQ	p_Proteobacteria;c_Gammaproteobacteria;o_Burkholderiales;f_Burkholderiaceae;g_LMUX01;s_
NGEE_A_bin19	JAMFSU000000000	MQ	p_Proteobacteria;c_Gammaproteobacteria;o_Burkholderiales;f_Burkholderiaceae;g_Herbaspirillum;s_
NGEE_A_bin2	JAMFST000000000	MQ	p_Myxococcota;c_Polyangia;o_Polyangiales;f_Polyangiaceae;g_;s_
NGEE_A_bin20	JAMFSS000000000	HQ	p_Proteobacteria;c_Alphaproteobacteria;o_Micavibrionales_A;f_UBA9219;g_JABDFY01;s_
NGEE_A_bin21	JAMFSR000000000	HQ	p_Acidobacteriota;c_Acidobacteriae;o_Acidobacteriales;f_Acidobacteriaceae;g_Granulicella_A;s_
NGEE_A_bin22	JAMFSQ000000000	MQ	p_Acidobacteriota;c_Acidobacteriae;o_Acidobacteriales;f_Acidobacteriaceae;g_Granulicella_C;s_
NGEE_A_bin23	JAMFSP000000000	MQ	p_Proteobacteria;c_Gammaproteobacteria;o_Pseudomonadales;f_Spongiibacteraceae;g_;s_
NGEE_A_bin24	JAMFSO000000000	MQ	p_Proteobacteria;c_Gammaproteobacteria;o_Steroidobacterales;f_Steroidobacteraceae;g_;s_
NGEE_A_bin25	JAMFSN000000000	HQ	p_Actinobacteriota;c_Actinomycetia;o_Mycobacteriales;f_Frankiaceae;g_;s_
NGEE_A_bin26	JAMFSM000000000	MQ	p_Myxococcota;c_Polyangia;o_Haliangiales;f_Haliangiaceae;g_;s_
NGEE_A_bin27	JAMFSL000000000	MQ	p_Bacteroidota;c_Bacteroidia;o_Chitinophagales;f_Chitinophagaceae;g_Puia;s_
NGEE_A_bin28	JAMFSK000000000	MQ	p_Proteobacteria;c_Gammaproteobacteria;o_Burkholderiales;f_Burkholderiaceae;g_Pararobbsia;s_
NGEE_A_bin29	JAMFSJ000000000	MQ	p_Verrucomicrobiota;c_Verrucomicrobiae;o_Pedosphaerales;f_UBA11358;g_UBA11358;s_
NGEE_A_bin3	JAMFSI000000000	MQ	p_Proteobacteria;c_Alphaproteobacteria;o_Sphingomonadales;f_Sphingomonadaceae;g_Novosphingobium;s_
NGEE_A_bin30	JAMFSH000000000	MQ	p_Actinobacteriota;c_Actinomycetia;o_Mycobacteriales;f_Mycobacteriaceae;g_Mycobacterium;s_
NGEE_A_bin31	JAMFSG000000000	MQ	p_Verrucomicrobiota;c_Verrucomicrobiae;o_Pedosphaerales;f_UBA11358;g_UBA11358;s_
NGEE_A_bin32	JAMFSF000000000	MQ	p_Bacteroidota;c_Bacteroidia;o_Chitinophagales;f_Chitinophagaceae;g_JABDFW01;s_
NGEE_A_bin33	JAMFSE000000000	MQ	p_Proteobacteria;c_Alphaproteobacteria;o_Acetobacterales;f_Acetobacteraceae;g_;s_
NGEE_A_bin34	JAMFSD000000000	MQ	p_Verrucomicrobiota;c_Verrucomicrobiae;o_Opitutales;f_Opitutaceae;g_CAIOZY01;s_
NGEE_A_bin35	JAMFSC000000000	MQ	p_Acidobacteriota;c_Acidobacteriae;o_Acidobacteriales;f_Acidobacteriaceae;g_Granulicella_C;s_
NGEE_A_bin36	JAMFSB000000000	MQ	p_Proteobacteria;c_Gammaproteobacteria;o_Burkholderiales;f_Burkholderiaceae;g_Paraburkholderia;s_Paraburkholderia phenazinium_C
NGEE_A_bin37	JAMFSA000000000	HQ	p_Bacteroidota;c_Bacteroidia;o_Sphingobacteriales;f_Sphingobacteriaceae;g_Mucilaginibacter;s_
NGEE_A_bin38	JAMFRZ000000000	MQ	p_Proteobacteria;c_Alphaproteobacteria;o_Rhizobiales;f_Xanthobacteraceae;g_Bradyrhizobium;s_Bradyrhizobium sp009766005
NGEE_A_bin39	JAMFRY000000000	MQ	p_Proteobacteria;c_Alphaproteobacteria;o_Acetobacterales;f_Acetobacteraceae;g_;s_
NGEE_A_bin4	JAMFRX000000000	MQ	p_Bacteroidota;c_Bacteroidia;o_Chitinophagales;f_Chitinophagaceae;g_Puia;s_
NGEE_A_bin40	JAMFRW000000000	MQ	p_Proteobacteria;c_Gammaproteobacteria;o_Burkholderiales;f_Burkholderiaceae;g_Rhizobacter;s_
NGEE_A_bin41	JAMFRV000000000	MQ	p_Proteobacteria;c_Gammaproteobacteria;o_Pseudomonadales;f_Pseudomonadaceae;g_Pseudomonas_E;s_
NGEE_A_bin42	JAMFRU000000000	MQ	p_Proteobacteria;c_Alphaproteobacteria;o_Acetobacterales;f_Acetobacteraceae;g_Acidocella;s_
NGEE_A_bin43	JAMFRT000000000	HQ	p_Actinobacteriota;c_Actinomycetia;o_Mycobacteriales;f_Frankiaceae;g_Frankia;s_Frankia sp902806485
NGEE_A_bin44	JAMFRS000000000	MQ	p_Bacteroidota;c_Bacteroidia;o_Sphingobacteriales;f_Sphingobacteriaceae;g_Mucilaginibacter;s_
NGEE_A_bin45	JAMFRR000000000	MQ	p_Actinobacteriota;c_Actinomycetia;o_Streptosporangiales;f_Streptosporangiaceae;g_Trebonia;s_
NGEE_A_bin5	JAMFRQ000000000	MQ	p_Proteobacteria;c_Gammaproteobacteria;o_Steroidobacterales;f_Steroidobacteraceae;g_Bog-1198;s_
NGEE_A_bin6	JAMFRP000000000	MQ	p_Bacteroidota;c_Bacteroidia;o_Sphingobacteriales;f_Sphingobacteriaceae;g_Mucilaginibacter;s_
NGEE_A_bin7	JAMFRO000000000	MQ	p_Proteobacteria;c_Alphaproteobacteria;o_Acetobacterales;f_Acetobacteraceae;g_Acidocella;s_
NGEE_A_bin8	JAMFRN000000000	MQ	p_Proteobacteria;c_Gammaproteobacteria;o_Nevskiales;f_Nevskiaceae;g_Nevskia;s_
NGEE_A_bin9	JAMFRM000000000	MQ	p_Bacteroidota;c_Bacteroidia;o_Sphingobacteriales;f_Sphingobacteriaceae;g_Mucilaginibacter;s_Mucilaginibacter sp014200495

a HQ, high quality draft; MQ, medium quality draft by MIMAG standards.
